# The delirium screening tool 4AT in routine clinical practice: prediction of mortality, sensitivity and specificity

**DOI:** 10.1007/s41999-021-00489-1

**Published:** 2021-04-04

**Authors:** Sigurd Evensen, Anette Hylen Ranhoff, Stian Lydersen, Ingvild Saltvedt

**Affiliations:** 1 Department of Neuromedicine and Movement Science, Faculty of Medicine and Health Sciences, Norwegian University of Science and Technology (NTNU), N-7491 Trondheim, Norway; 2 Department of Medicine, Diakonhjemmet Hospital, Oslo, Norway; 3 Department of Clinical Science, University of Bergen, Bergen, Norway; 4 Regional Centre for Child and Youth Mental Health and Child Welfare, Department of Mental Health, Norwegian University of Science and Technology (NTNU), Trondheim, Norway; 5 Department of Geriatrics, Clinic of Medicine, St. Olavs Hospital, Trondheim University Hospital, Trondheim, Norway

**Keywords:** Delirium, Delirium screening, 4AT, Geriatrics

## Abstract

**Purpose:**

Delirium is common and associated with poor outcomes, partly due to underdetection. We investigated if the delirium screening tool 4 A’s test (4AT) score predicts 1 year mortality and explored the sensitivity and specificity of the 4AT when applied as part of a clinical routine.

**Methods:**

Secondary analyses of a prospective study of 228 patients acutely admitted to a Medical Geriatric Ward. Physicians without formal training conducted the index test (the 4AT); a predefined cut-off ≥ 4 suggested delirium. Reference standard was delirium diagnosed by two geriatricians using the *Diagnostic and Statistical Manual of Mental Disorders 5 (DSM-5)*. We calculated hazard ratios (HR) using Cox regression based on the groups 4AT = 0, 1–3, 4–7 and ≥ 8, first unadjusted, then adjusted for the covariates age, comorbidity, and personal activities of daily living. We calculated sensitivity, specificity, and the area under the receiver operating curve (AUC).

**Results:**

Mean age of patients was 86.6 years, 139 (61.0%) were female, 78 (34.2%) had *DSM-5* delirium; of these, 56 had 4AT-delirium. 1 year mortality was 27.6% (63 patients). Compared to 4AT score 0, the group 4AT ≥ 8 had increased 1 year mortality (HR 2.86, 95% confidence interval 1.28–6.37, *p* = 0.010). The effect was reduced in multiadjusted analyses (HR 1.69, 95% confidence interval 0.70–4.07, *p* = 0.24). Sensitivity, specificity, and AUC were 0.72, 0.84, and 0.88, respectively.

**Conclusions:**

4AT ≥ 8 indicates increased mortality, but the effect was reduced in multiadjusted analyses. 4AT had acceptable sensitivity and specificity when applied as a clinical routine.

## Key summary points

**Aim:**

Investigate if 4AT score predicts 1 year mortality and explore the sensitivity and specificity of the 4AT when applied as part of a clinical routine.

**Findings:**

4AT score is one of several clinical characteristics predicting 1 year mortality. The 4AT has reasonable sensitivity and specificity to detect delirium in a clinical routine setting.

**Message:**

The 4AT seems to be a useful tool for delirium screening and may predict mortality.

## Background

Delirium is an acute disturbance in attention, awareness and cognition occurring secondary to acute illness, trauma or surgery [1]. Delirium is common in all hospital settings, affects about 30% of hospitalized older patients and is associated with elevated risk of death, dementia, and institutionalization [2–4]. The costs of delirium are substantial [5]. Older age, cognitive impairment, comorbidity and frailty are the most significant risk factors [6, 7].

Although common, delirium is underdiagnosed in as many as 60% of cases [8], and underdetection may contribute to poor outcomes [9]. Screening for delirium is, therefore, recommended in hospitalized older patients [2, 4]. The 4 A’s test (4AT) is a short screening tool for delirium and cognitive impairment developed in the UK [10]. It has been translated into several languages, and validation studies report high sensitivity and specificity for the diagnosis of delirium [11–18]. A recent meta-analysis including 3702 patients concluded that the 4AT has a sensitivity and specificity of 88% for delirium screening [19].

The authors of the 4AT claim that health care professionals using the 4AT need no formal training [10]. One study investigated sensitivity and specificity of the 4AT when performed by nurses [20], but to our knowledge, no studies have investigated sensitivity and specificity of the 4AT when performed by physicians outside strict validation studies. Two recent papers indicate that 4AT score predict in-hospital mortality [21, 22], but it is unknown whether 4AT score may predict long-term mortality. The aim of this paper is (1) to investigate whether total 4AT score predicts 1 year mortality in hospitalized geriatric patients and (2) to explore the sensitivity and specificity of the 4AT for diagnosing delirium when applied as part of a clinical routine by physicians without formal training in scoring the 4AT.

## Methods

This paper reports secondary analyses of data collected in a project on delirium motor subtypes (DeMo) [23–25] and follows the reporting practice recommended in the Standards for Reporting of Diagnostic Accuracy Studies (STARD) [26]. The Regional Committee for Medical and Health Research Ethics of Mid-Norway approved the study (REK Central 2015/474) which was conducted according to the standards in the Declaration of Helsinki.

### Settings and participants

Consecutive patients ≥ 75 years who were acutely admitted to the medical geriatric ward at St. Olavs Hospital, Trondheim University Hospital, Norway, between May 2015 and January 2017 were eligible for inclusion. The only exclusion criteria were inability to speak/read Norwegian and previous participation in the study, and no patients were excluded from the DeMo-study or this sub-study due to sensory impairments, functional status, severity of acute disease or cognitive impairment as is often the case in studies on geriatric patients. Patients could consent to participation if considered to have the capacity to do so; for those without the capacity, a proxy could sign the consent form.

St Olavs Hospital is a university hospital with 1000 beds, serving as a tertiary hospital for the 455,000 inhabitants of the region Trøndelag and as local hospital for the 200,000 inhabitants in the city of Trondheim and four nearby municipalities. The acute geriatric ward has 15 single-bed rooms and is an integrated part of the medical department, mean length of stay during the study period was 7.6 days. Most patients arrive as acute admissions from the emergency department, a substantial number arrive as transfers from other departments. Patients receive comprehensive geriatric care [27] from a team of physicians, nurses, physiotherapists, and occupational therapists, all integrated at the ward [23–25].

### Index test—the 4AT

**Fig. 1:**
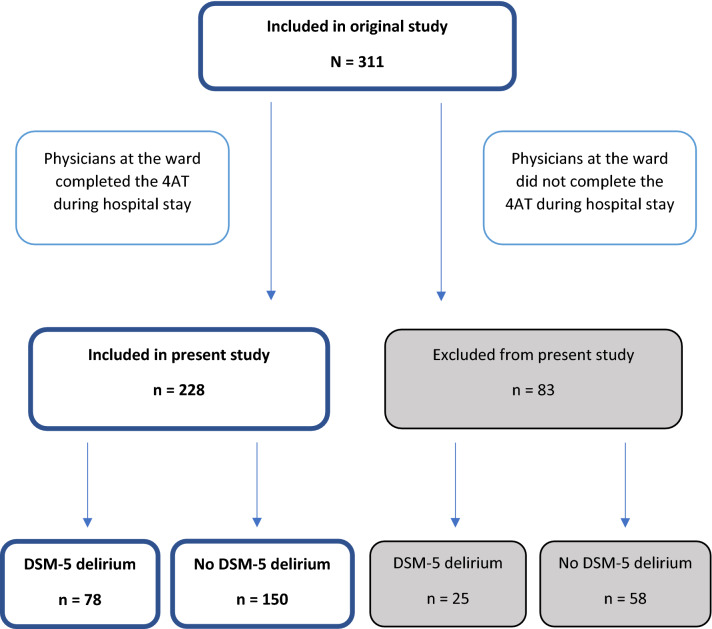
Flowchart illustrating patients included in the original study and the present study

Parallel to the DeMo project, we sought to implement the 4AT in the daily routine at the ward to increase the focus on delirium among the staff and improve the diagnostic work-up for delirium. Physicians at the ward, four specialists in geriatric medicine and four residents in training for geriatric medicine, completed the Norwegian version of the 4AT [28] the first day after the patient’s arrival. The physicians were encouraged to assess all new patients admitted to the ward including those participating in the DeMo project consecutively but were not reminded about the 4AT if they did not complete the test after the first day. Figure 1 illustrates the inclusion process. The physicians were given brief information about the DeMo project and the 4AT but did not receive formal training in scoring the 4AT. All clinical information was available to the physicians completing the index test.

The 4AT contains four items: (1) a bedside evaluation of alertness; (2) the Abbreviated Mental Test 4 (AMT4); (3) the months of the year backwards (MOTYB) attention task, and (4) an evaluation of recent acute changes or fluctuations in mental status. Item 1 is scored 0 for normal alertness and 4 for altered alertness. Items 2 and 3 are scored 0 to 2. Item 4 is scored 0 if acute changes or fluctuations in mental status are not present, and 4 if such changes are present. The maximum total score is 12 [18]. We used the same predefined cut-off scores as other validation studies; a score of 0 indicates no cognitive problems, a score of 1–3 indicates cognitive impairment and a score ≥ 4 indicates ongoing delirium [11–18].

### Reference standard

The reference standard diagnosis was delirium according to the *Diagnostic and Statistical Manual of Mental Disorders 5* (*DSM-5)* criteria [1]. The assessment was completed by the first author and a now retired professor in geriatric medicine. The first author visited new patients and assessed awareness and alertness based on items from the Memorial Delirium Assessment Scale (MDAS) [29], tested attention by use of the digit span and tested cognitive function by use of the orientation and memory items from the MDAS. As a supplement, the first author did chart review and interviewed nurses, doctors, and proxies about signs of delirium. Then, the two assessors discussed all cases, considering all available information from the entire hospital stay when deciding if the patient filled the DSM-5 criteria for delirium or not.

The 4AT score was not used in the reference standard assessment, but the assessors were not strictly blinded to the result of the index test. The result of the reference standard assessment was not available to the ward’s physicians.

### Data collection

Descriptive data were collected prospectively and were based on all available information from hospital records and interviews with patients and proxies. We used the Global Deterioration Scale (GDS) as a measure of pre-hospital cognitive function [30]. The GDS ranges from 1 to 7, 1 indicating no cognitive problems and 7 representing end-stage dementia. We defined dementia as a score of ≥ 4, since a score of 3 describes mild cognitive impairment and a score of 4 describes mild dementia [30]. We used the Barthel Index (BI) based on interviews with proxies as a measure of pre-hospital function in personal activities of daily living (pADL) [31]. BI ranges from 0 to 20; an increasing score indicates independency in pADL. We used the Cumulative Illness Rating Scale (CIRS) as a measure of comorbidity. CIRS ranges from 0 to 56; an increasing score illustrates increasing comorbidity and is associated with elevated mortality [32]. We used a modified APACHE II score as a measure of severity of acute illness [33]. APACHE II ranges from 0 to 71; an increasing score indicates elevated level of organ failure. Data on 1 year mortality were collected from the hospital records, which are synchronized with the National Death Registry.

### Statistical analyses

We report continuous data as means and standard deviations (SD) and dichotomous data as numbers and percentages. To evaluate the association between 4AT score and 1 year mortality rate we constructed Kaplan–Meier plots based on groups consisting of a 4AT score of 0, 1–3, 4–7 and ≥ 8. We calculated hazard ratios (HR) using Cox proportional hazards regression analyses based on the same groups. The choice of these groups were based on an assumption that patients with no signs of cognitive impairment (4AT = 0) would have the best prognosis, followed by patients with cognitive impairment without delirium (4AT 1–3) and patients with delirium (4AT ≥ 4). Further, we wanted to explore if very high scores (4AT ≥ 8) indicated particularly poor prognosis, as we presumed that a very high 4AT-score would indicate a more certain delirium. HR was calculated unadjusted, then adjusted one at a time and simultaneously for age, CIRS, and BI, since these variables have prognostic impact in older adults [34, 35]. The ability of the 4AT to discriminate between patients with and without delirium was examined using the area (AUC) under the receiver operating characteristic curve (ROC). We calculated sensitivity and specificity for the 4AT as a screening test for delirium with a cut-off of ≥ 4, including Wilson Score confidence intervals. We report 95% confidence intervals (CI) where relevant, and regard two-sided *p*-values under 0.05 to indicate statistical significance. Analyses were carried out in SPSS 25, StatXact 11, and Stata 15.

## Results

In total, 311 patients were included in the DeMo project. Physicians at the ward completed the 4AT in 228 patients (73.3%), who constitute the sample reported here. Of these, 139 (61.0%) were women, 117 (51.3%) had dementia, and 218 (95.6%) were home-living. Mean length of stay for patients with delirium was 10.3 (SD 6.9) days. Seventy-eight patients (34.2%) had delirium according to the *DSM-5* criteria, 80 (35.1%) had delirium according to the 4AT, and 63 (27.6%) died during the 1 year follow-up. Table 1 shows the number of patients, baseline characteristics, *DSM-5* delirium, and 1 year mortality rates in the different groups of 4AT scores.

**Table 1:** Baseline characteristics, *DSM-5* delirium, and 1 year mortality for the entire study population and the subgroups of 4AT scores of 0, 1–3, 4–7, and 8–12

	4AT score				All	*p* value^a^
	0	1–3	4–7	8–12		
Number (%)	83 (36.4)	65 (28.5)	61 (26.7)	19 (8.3)	228 (100)	
Age Mean (SD)	85.7 (5.2)	87.2 (5.1)	87.2 (5.2)	86.1 (5.1)	86.6 (5.2)	0.23
GDS^b^ (1–7) Mean (SD)	2.3 (1.4)	3.7 (1.6)	4.2 (1.4)	4.4 (1.1)	3.4 (1.7)	< 0.001*
Barthel Index^c^ (0–20) Mean (SD)	17.5 (2.9)	15.9 (3.5)	15.7 (3.9)	13.8 (5.1)	16.3 (3.7)	< 0.001*
CIRS^d^ (0–56) Mean (SD)	12.4 (4.3)	14.0 (4.4)	12.8 (4.2)	15.8 (5.1)	13.2 (4.4)	0.03*
APACHE-II^e^ (0–71) Mean (SD)	9.3 (3.1)	9.3 (2.8)	9.2 (2.3)	9.2 (2.8)	9.3 (2.8)	0.83
Dementia^f^ (%)	16 (19.3)	42 (64.6)	44 (72.1)	15 (78.9)	117 (51.3)	< 0.001*
*DSM*^g^*-5* delirium (%)	2 (2.4)	20 (30.8)	39 (63.9)	17 (89.5)	78 (34.2)	< 0.001*
1 year mortality (%)	18 (21.7)	20 (30.8)	16 (26.2)	9 (47.4)	63 (27.6)	0.011*

*p < 0.05

^a^*p* values were calculated using Cox regression models for 1 year mortality, a Cochran Armitage test for trend, and linear regression for continuous variables

^b^*GDS* Global Deterioration Scale

^c^Barthel Index before admission

^d^*CIRS* Cumulative Illness Rating Scale, total score

^e^*APACHE-II* Acute Physiology and Chronic Health Evaluation

^f^Dementia defined as GDS score ≥ 4

^g^*DSM* Diagnostic and Statistical Manual of Mental Disorders

**Fig. 2:**
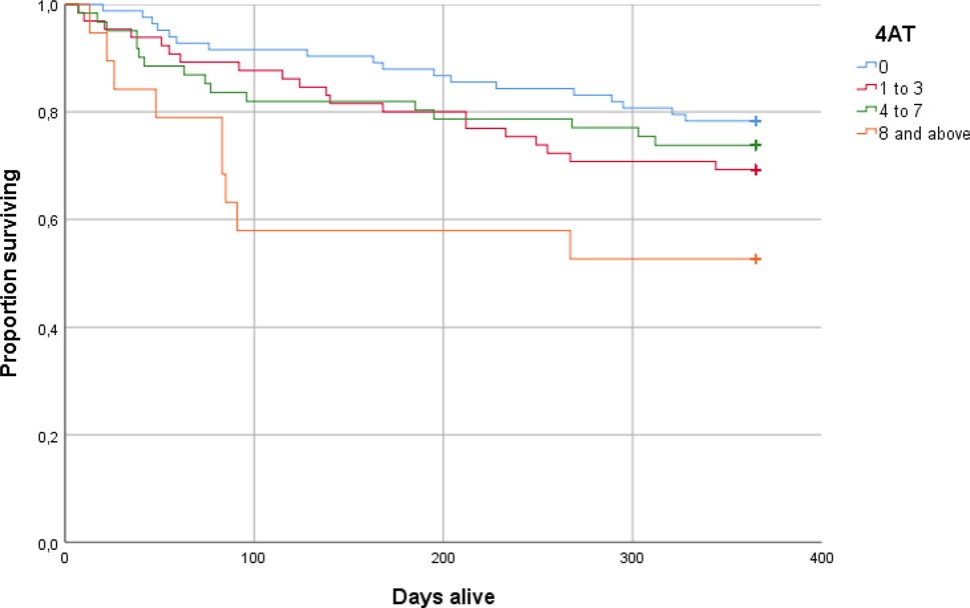
Kaplan–Meier survival curves for the four categories of 4AT score

Figure 2 illustrates the Kaplan–Meier survival curves based on the four categories of 4AT score. The figure indicates that the 4AT categories 1–3 and 4–7 have somewhat lower survival than category 0, and that those with a 4AT score of ≥ 8 seem to have substantially lower survival. Compared to 4AT = 0, HR for the group 4AT = 1–3 was 1.52 (95% CI 0.80–2.87, *p* = 0.20), HR for the group 4AT = 4–7 was 1.30 (95% CI 0.66–2.54, *p* = 0.45) and HR for the group 4AT ≥ 8 was 2.86 (95% CI 1.28–6.37, *p* = 0.010). Adjusting for covariates did not change the effect for the groups 4AT = 1–3 and 4–7 substantially but reduced the effect in the group 4 AT ≥ 8, (HR = 1.69, 95% CI 0.70–4.07, *p* = 0.24).

**Fig. 3:**
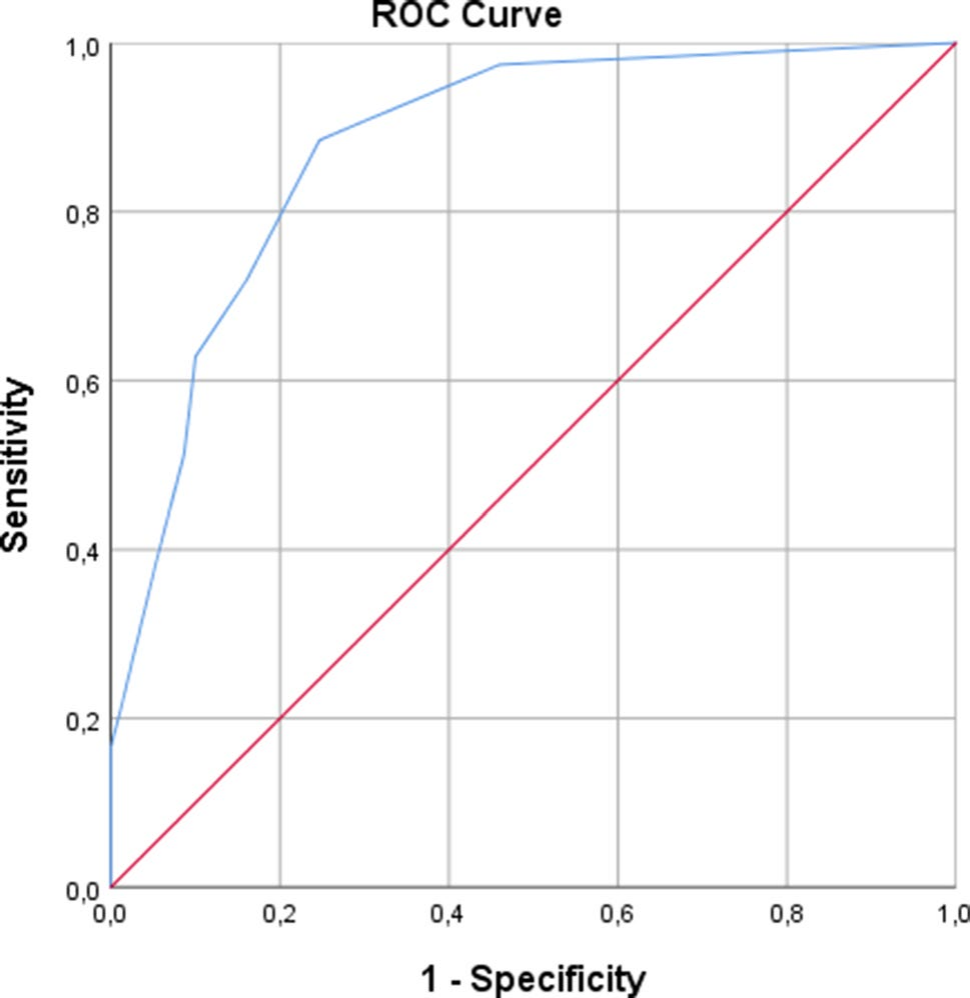
Receiver operating characteristic curve (ROC) for the 4AT as predictor of *DSM-5* delirium

Figure 3 shows the ROC curve. The estimated area AUC under the ROC curve was 0.88 (CI 0.83–0.92). Among the 78 patients with *DSM-5* delirium, 56 (71.8%) had a 4AT score of ≥ 4 and 22 (28.2%) had a 4AT score of ≤ 3, giving a sensitivity of 0.72 (CI 0.61–0.81). Among the 150 patients without *DSM-5* delirium, 126 (84%) had a 4AT score of ≤ 3, giving a specificity of 0.84 (CI 0.77–0.89).

In total, 14 (6.1%) patients were given a score of 4 on item 1 and 76 (33.3%) were given a score of 4 on item 4. All patients with a score of 4 on item 1 had *DSM-5* delirium, whereas 54 (71.1%) of the 76 patients with a score of 4 on item 4 had *DSM-5* delirium. Among patients with *DSM-5* delirium, 24 out of 78 (30.8%) were given a score of 0 on item 4.

## Discussion

In this study on acutely admitted geriatric patients, a 4AT score of ≥ 8 predicted 1 year mortality in unadjusted analyses, but this effect was reduced in multivariate analyses. The 4AT had reasonable sensitivity and good specificity for detecting delirium, even when performed by physicians without formal training working in a routine clinical setting outside a strict validation study.

These results indicate that a 4AT score of ≥ 8 predicts poor outcomes; the 1 year mortality in this group was close to 50%. In this study, 89.5% of the patients with a 4AT score of ≥ 8 had *DSM-5* delirium, and in view of previous studies [3], we believe our results simply indicate that geriatric patients with manifest delirium have increased risk of mortality. A challenge is to identify the patients with delirium who have particularly poor outcomes, and a systematic review indicate that older patients with long-lasting delirium, hypoactive subtype, high delirium severity and comorbid dementia, and depression have poorer outcomes [35]. Recent studies have documented that a high 4AT score indicates poor outcomes included elevated short-time mortality [21, 22]. Our results indicate that the 4AT may predict long-term mortality as well, but that other factors like age, comorbidity and particularly functional status also provide important information about prognosis in geriatric patients [34].

Validation studies have demonstrated high sensitivity and specificity of the 4AT as a delirium screening tool [19], but to our knowledge, this paper is the first reporting sensitivity and specificity of the 4AT when conducted by physicians outside strict validation studies; the reasonable sensitivity and specificity we report support the use of 4AT as a delirium screening tool. Still, our material raises concerns about items 1 and 4. Item 1 addresses alertness, a core feature of delirium. In our sample, only 14 patients (6.1%) received a score of 4 on item 1. This is remarkably few, since 78 patients (34.2%) had delirium according to the DSM-5 criteria, and results may indicate that physicians, even geriatricians and residents training for geriatric medicine, do not fully understand the concept of alertness. Since the index test and the reference standard were not performed strictly within a limited time interval, an alternative explanation is that an altered level of alertness was not present when the index test was performed and was obvious when reference assessment was completed. Further, the physicians missed acute changes and/or fluctuations in mental status in 24 out of the 78 patients (30.8%) with DSM-5 delirium, which may indicate that item 4 is more frequently underscored in real-life settings than in validation studies. Reasons for underscoring may be that 4AT assessors, who were busy clinicians, did not have proxies available or did not find the time to talk to proxies and nurses when completing item 4 and consequently missed important information about acute onset and fluctuation. Another possibility may be that the assessors did not notice and emphasize the information about fluctuations. A recent review and meta-analysis of diagnostic accuracy of the 4AT requests studies identifying training needs, and we believe our findings indicate that health care staff should have formal training before using the 4AT, with a focus on how to score items 1 and 4 [19, 20].

More than half of the patients described in this paper had dementia according to our definition (GDS ≥ 4). Since the DeMo project was designed with a pragmatic diagnostic approach to dementia, our data should not be used to validate the 4AT in patients with dementia. Still, Table 1 indicates some interesting trends. First, 19.3% of those scoring 0 on the 4AT have dementia according to our definition. Further, dementia is more common in those with a 4AT score indicating delirium than those with a 4AT score indicating cognitive impairment but not ongoing delirium. This is in line with previous studies finding that dementia is a strong risk factor for delirium [6] and may indicate that patients with delirium with no known dementia diagnosis should be examined for dementia later. Table 1 may also indicate increasing levels of comorbidity and lower pADL-function with an increasing 4AT score, raising the question as to whether the 4AT could be used to identify vulnerable patients in a broader sense than just cognitive impairment, for instance patients with functional impairment and risk of falls.

The major limitations of the study are the design with secondary analyses and that the index test and the reference standard were not strictly conducted within a limited period, introducing the possibility that the patients could be free of delirium symptoms when the index test was conducted but have more pronounced symptoms later. These limitations could be an explanation to the concerns discussed about item 1 and 4 of the 4AT, but we still believe that these concerns are valid since both tests were done within a relatively short timeframe of the same hospitalization. Further, sensitivity could be higher than 0.72 if the reference assessment had been conducted immediately after the index test. Other limitations include the fact that the assessors of the reference standard were not strictly blinded to the results of the index test and that the first author on a general basis occasionally supervised junior doctors on how to score the 4AT, introducing a possible bias. Our approach to diagnose dementia was pragmatic, and the data on 4AT score and dementia must be interpreted carefully. Our sample consists of frail geriatric patients, so results may not be generalizable to fit hospitalized adults. The fact that the study was not conducted as a strict validation study could also be considered as a strength since we can report how the 4AT performs as a delirium screening tool in a real-life setting. The completeness of data and the long follow-up are other strengths of the study.

To conclude, this study on acutely admitted geriatric patients demonstrated that a high 4AT score may indicate elevated 1 year mortality, although age, comorbidity, and pADL-function also provide information about mortality risk. The 4AT seems to have reasonable sensitivity and specificity as a delirium screening tool when applied as part of a clinical routine outside validation studies. Still, our results may indicate that health care professionals should get formal training before using the 4AT and that such training may improve the sensitivity of the 4AT for delirium screening.

## Data Availability

The dataset is not publicly available because the consent did not include a consent to share data or make data publicly available.
